# Effect of replacing inorganic minerals with small peptide chelated minerals on production performance, some biochemical parameters and antioxidant status in broiler chickens

**DOI:** 10.3389/fphys.2022.1027834

**Published:** 2022-10-18

**Authors:** Jing Kong, Ting Qiu, Xia Yan, Lili Wang, Zhiyong Chen, Gengsheng Xiao, Xin Feng, Huihua Zhang

**Affiliations:** ^1^ School of Life Science and Engineering, Foshan University, Foshan, China; ^2^ Laboratory of Livestock and Poultry Breeding, Institute of Animal Science, Guangdong Academy of Agricultural Sciences, Guangzhou, China; ^3^ Guangdong Xingtengke Biotechnology Co., Ltd., Zhaoqing, China

**Keywords:** inorganic trace mineral, small peptide chelate mineral, growth performance, antioxidant status, tibia mineral content, fecal mineral content

## Abstract

Due to the low bio-availability of inorganic trace minerals, its application in poultry production has been causing many problems such as environment pollution and waste of resources. The current study was designed to evaluate if replacing inorganic trace minerals (ITM) with small peptide chelate trace minerals (SPM) affects production performance, some biochemical parameters and antioxidant status, tibia mineral deposition, and fecal mineral content in 817 white-feathered broilers. A total of 432 broilers (21-day-old) were randomly divided into four groups with six replicates of 18 chicks each. The four groups included inorganic trace minerals group (addition of 1,000 mg/kg ITM; common practice by commercial poultry farms), three organic trace minerals groups with supplementation of 150, 300, and 500 mg/kg SPM, respectively. The experiment lasted for 30 days. The results showed that there was no significant difference in growth performance and slaughter performance among the four groups (*p* > 0.05). Total cholesterol in the SPM group was significantly lower than those in the ITM groups (*p* < 0.01). Compared with the ITM group, the serum urea nitrogen in 150 and 300 mg/kg SPM groups decreased significantly (*p* < 0.01). Among all SPM treatments, 300 mg/kg SPM groups had the highest serum glutathione peroxidase (GSH-Px) activity (*p* < 0.01). The activity of copper and zinc superoxide dismutase (Cu/Zn SOD) of liver in ITM group was the lowest among the four groups (*p* < 0.01). The catalase (CAT) activity of liver in the 150 mg/kg SPM group was significantly higher than the ITM group and 300 mg/kg SPM group (*p* < 0.05). Compared to the ITM group, the iron content of the tibia was significantly increased in 300 mg/kg SPM group (*p* < 0.05) and 500 mg/kg SPM group (*p* < 0.01). Compared to the ITM group, dietary supplementation with SPM significantly reduced fecal content of zinc and manganese (*p* < 0.01). The 150 mg/kg SPM and 300 mg/kg SPM group had significantly reduced content of iron (*p* < 0.05). This study demonstrated that replacing inorganic minerals with low doses of SPM (300 and 500 mg/kg) did not negatively affect growth and slaughter performance, as well as the antioxidant status of broiler chickens. In addition, SPM can also promote mineral content in the tibia and reduce mineral content in the feces.

## Introduction

Trace elements such as copper, iron, zinc, and manganese are essential nutrients for maintaining animal life and growth. These elements are directly or indirectly involved in the physiological and biochemical processes of the animal body (Richards et al., 2010). Their deficiency can cause specific symptoms such as reduced feed intake, growth retardation and even death (Liu et al., 2014). Copper, zinc, manganese, and selenium are cofactors of metalloenzymes (superoxide dismutase, SOD), catalase (CAT), and glutathione peroxidase (GSH-Px), playing an essential role in the antioxidant system and lipid peroxidation (Barandier et al., 1999; Ghasemi et al., 2020). In practice, trace minerals are inexpensive and readily available. Producers have been using higher mineral safety margins in commercial corn-soyean-based rations for the past 20 years by adding high levels of minerals to meet the nutritional needs of birds (Bao and Choct, 2009). Traditionally trace minerals have been added to commercial broiler rations in the form of inorganic salts such as sulfates, oxides, carbonates, and phosphates. As a result, excessive minerals are excreted to the environment, causing many problems, such as soil contamination, potential toxicity due to excessive accumulation, reduced crop yields, and waste of resources (Giordano et al., 1975). Trace minerals in the form of inorganic salts usually have poor bio-availability (Mézes et al., 2012). For these reasons, it is imperative to find minerals replacements with higher bio-availability.

Organic trace minerals have higher bio-availability for animals than inorganic trace minerals (ITM) due to its better chemical stability and physical heterogeneity of the chelate form (Ghasemi et al., 2020). Organic trace minerals are absorbed through the amino acid or peptide transport system, which is the main reason for the increased bio-availability (Muszyński et al., 2017). Stable organic trace elements can avoid the adsorption and precipitation of minerals by antagonistic factors (e.g., humic acid) in the intestinal lumen. Amino acid complexes or chelates are the animal organism’s main form of metal ion uptake and transport, as well as intermediates in the organism’s protein synthesis, they can reduce many biochemical processes, accelerate the absorption rate, and save physical energy consumption. Lower supplementation amount of organic minerals can also meet the nutritional needs of animals (Nollet et al., 2007; Richards et al., 2010).

Research has claimed that uric acid has a correlation with the antioxidant capacity of birds (Tsahar et al., 2006). Echeverry et al. (2016) showed that organic trace elements compared to inorganic supplements, lowered malondialdehyde concentrations and increased plasma uric acid, thus reducing oxidative stress. It also reduced the excretion of mineral in feces and improved mineral deposition in tissues (Wang et al., 2018). In addition, higher serum glutathione peroxidase and superoxide dismutase activities in serum were also observed with organic micro-nutrient treatment (Ghasemi et al., 2020). Low dose glycine mineral complex can improve the apparent bio-availability of iron, manganese, and zinc (Sun et al., 2020). In previous studies, the effects of different types of amino acid chelates on birds were investigated. Organic supplements in the form of small peptides, which are rarely reported, are chelates of soybean peptides with inorganic salts. Low levels of small peptide metal chelates, with no adverse effects on growth performance and health status of weaned piglets, was reported to significantly reduce copper emissions (Xu et al., 2018).

Few effects of small peptide metal chelates on avian species have been reported. Therefore the objective of this study was to evaluate the effect of low levels of small peptide chelated minerals (SPM) on production performance, blood characteristics and antioxidant status, liver antioxidant status, fecal mineral content, and tibial mineral deposition in commercial 817 white-feather broilers. The results of the current study would provide references for application of small peptide chelated mineral in broiler chickens.

## Materials and methods

### Experimental animals

A total of 432 21-day-old (438 ± 8.76 g) 817 white feather broilers (hybrid of high-quality white-feathered fast giant sire breed cock and brown shell egg hen) were randomly divided into four groups with six replicates per group and 18 chickens per replicate. The four groups are: inorganic trace element addition group (1,000 mg/kg ITM), organic trace element groups with 150, 300, and 500 mg/kg SPM, respectively. All broilers were housed in 66 cm × 66 cm × 43 cm wire cages. The experiment lasted 30 days with two phases (Days 21–35 and 36–50). Ambient conditions such as temperature, lighting, and humidity were based on standard farm management practice. During the study, all the birds had free access to feed and clean water. This study was approved by the Animal Research Ethics Committee of Foshan University, China.

### Dietary treatments

The basal diet (Table 1) was formulated according to the National Research Council (1998) for corn-soybean -based diets, with appropriate adjustments according to Chinese agricultural industry standards (NY/T33-2004, Feeding Standard of Chicken. The Ministry of Agriculture of the People’s Republic of China, 2004). The doses of mineral supplementation in the four dietary treatments are shown in Table 2. The control group was the common practice used by commercial broilers farms (basal diet plus 1,000 mg/kg of ITM). The experimental groups were basal diet plus 150, 300, and 500 mg/kg of small peptide chelate minerals (SPM), respectively. The trace minerals are provided by XingTengke Biotechnology Co., Ltd. (Guangdong, China).

**TABLE 1:** Composition and nutrient level of the basic diet (as feeding basis).

Item	Starter (21–35 days)	Finisher 36–50 days
Ingredients		
Corn	58.49	57.66
Soybean meal	29.08	27.21
Corn protein powder	5	5
Stone Powder	1.36	1.22
DCP	1	1.04
L-lysine sulfate	0.49	0.39
DL-Methionine	0.27	0.21
NaCl	0.32	0.32
L-Threonine	0.1	0.06
Choline chloride (50%)	0.08	0.08
Vitamin and mineral premix^a^	0.35	0.35
Phytase	0.01	0.01
Sodium humate	0.1	0.15
Lard	3.35	6.3
Calculated nutrient level		
ME (MJ/kg)	12.8	13.52
CP	20.62	19.61
EE	6.04	8.87
CF	2.42	2.33
Ca	0.85	0.8
TP	0.55	0.54
Lys	1.34	1.21
Met	0.58	0.52
Met + Cys	0.86	0.78
Thr	0.86	0.79

^a^ Supplied per kilogram of diet: vitamin A 12,000 IU vitamin D3 3,000 IU, vitamin E 101 IU, vitamin K 32 mg, vitamin B11 mg, vitamin B23 mg, vitamin B62 mg, vitamin B12 0.01 mg, pantothenate 4 mg, nicotinic acid 20 mg, folic acid 0.5 mg, biotin 0.05 mg. Trace minerals are supplemented regarding the experimental design and added to the premix.

**TABLE 2:** Experimental treatment of broiler diets and supply of each trace element (mg/kg).

Item	Inorganic^a^	150 mg/kg SPM	Organic^b^	500 mg/kg SPM
	1,000 mg/kg ITM		300 mg/kg SPM	
Cu	6	0.9	1.8	3
Fe	30	2.25	4.5	7.5
Zn	50	6	12	20
Mn	60	9	18	30

^a^ Inorganic trace elements copper, iron, zinc, and manganese added to the diet are sulfates.

^b^ Organic trace elements copper, iron, zinc, and manganese added to the diet are small peptide chelates.

### Growth performance

Feed intake (FI) was monitored daily. Body weight (BW) was measured on the day 35 and 50 before morning feeding. Average daily feed intake (ADFI), body weight average daily gain (ADG), and feed conversion ratio (FCR) were calculated for each growth phase during the growth period (21–35, 35–50 and 21–50 days).

### Sample collection

On day 50, one bird with body weight close to the average weight per replicate was selected for harvesting blood samples and tissue collections. Briefly, blood samples were collected from the wing vein and sit for 2 h before separating the serum. After blood collection, the bird was sacrificed by cervical dislocation and exsanguinated. Then the liver was immediately removed, weighed, and stored in liquid nitrogen to determine liver antioxidant activity. Dressed weight, eviscerated weight, semi-eviscerated weight, breast muscle weight, leg muscle weight, and abdominal fat weight were measured. Dressing percentage, breast muscle percentage, leg muscle percentage, and abdominal fat percentage were calculated. The left tibia was removed for mineral analysis. The fecal samples were collected by cage during the last 3 days. Sample from each replicate was mixed thoroughly and dried at 65°C for 48 h in an oven.

### Serum and liver parameters analysis

Based on the liver sample weight, sterile, ice-cold saline (0.9%) was added (9:1), and the sample was then homogenized until there were no tissue clumps. The homogenates were centrifuged at 2,500 × g for 10 min at 4°C. The concentrations of albumin, alkaline phosphatase, total cholesterol, urea, and total protein in serum were detected using kits. Antioxidant indices including CAT, malondialdehyde (MDA), total antioxidant capacity (T-AOC), GSH-Px, and copper zinc superoxide dismutase (Cu/Zn-SOD) concentrations in liver and serum samples were analyzed according to the kit instructions. All kits were purchased from Nanjing Jiancheng Bioengineering Institute (Nanjing, Jiangsu, China).

### Minerals analysis

The dried fecal samples were crushed with a pulverizer equipped with a stainless steel blade and passed through a 1 mm sieve to prepare a homogeneous sample. The tibia samples were thawed and boiled in deionized water for 10 min. Soft tissues were stripped and fat was extracted from the sample with petroleum ether for 24 h. The samples were dried in an oven at 105°C for 12 h. The trace minerals content of the fecal and tibia samples were analyzed by flame atomic absorption spectrophotometer (AA-7000, Shimadzu, Japan).

### Statistical analysis

All data were analyzed by one-way ANOVA using SPSS 26.0 software (SPSS Inc., Chicago, IL, United States) with treatment as a fixed effect, followed by a post hoc Tukey procedure to test for significant differences between treatments. Graphs are generated by GraphPad Prism 9.4 software. The significance was declared at *p* < 0.05.

## Results

### Growth and slaughter performance

The result of broiler growth performance was presented in Table 3. The ADG, ADFI, and FCR among ITM and SPM groups were no significant differences (*p* > 0.05). The slaughter performance data of broilers on the day 50 were presented in Table 4. There were no significant differences in dressing percentage, semi-eviscerated weight, eviscerated weight, breast muscle percentage, leg muscle percentage and abdominal fat percentage of the experimental broilers among all groups (*p* > 0.05).

**TABLE 3:** Effect of supplementation of ITMs and SPMs in basal rations on growth performance of chickens.

Item	1,000 mg/kg ITM	150 mg/kg SPM	300 mg/kg SPM	500 mg/kg SPM	SEM	*p*-value
Body weight						
Day 21, g	438.54	438.54	438.54	438.54	0.52	1.00
Day 35, g	1,060.07	1,067.71	1,074.56	1,067.19	7.81	0.91
Day 50, g	1,859.80	1,881.03	1,849.64	1,902.16	23.02	0.85
Starter stage (day 21–35)						
ADG, g	41.44	41.95	42.4	41.91	0.51	0.93
ADFI, g	77.26	76.14	76.46	76.08	0.86	0.96
FCR	1.87	1.82	1.81	1.82	0.01	0.42
Finisher stage (day 36–50)						
ADG, g	53.32	54.22	51.67	55.66	1.20	0.70
ADFI, g	113.66	121.62	115.9	125.3	3.07	0.54
FCR	2.14	2.26	2.25	2.25	0.05	0.76
Total stage (day 21–50)						
ADG, g	47.38	48.08	47.04	48.79	0.77	0.86
ADFI, g	94.59	99.64	96.18	100.28	1.82	0.65
FCR	2.00	2.08	2.05	2.06	0.02	0.68

**TABLE 4:** Effect of supplementation of ITMs and SPMs in basal rations on slaughter performance of chickens.

Item	1,000 mg/kg ITM	150 mg/kg SPM	300 mg/kg SPM	500 mg/kg SPM	SEM	*p*-value
Dressing percentage (%)	90.19	87.79	89.14	88.25	0.33	0.08
Semi-eviscerated weight, g	1,415.6	1,401.17	1,404.5	1,447.5	11.47	0.50
Eviscerated weight, g	1,211.4	1,202.33	1,199.17	1,246	7.13	0.13
Breast muscle percentage (%)	20.13	20.48	20.43	19.42	0.46	0.85
Leg muscle percentage (%)	19.79	21.51	20	20.43	0.41	0.46
Abdominal fat percentage (%)	2.67	2.56	3.35	3.14	0.15	0.30

### Serum and liver parameters

Serum concentration of total cholesterol of ITM group broilers was significantly higher than those of SPM groups (*p* < 0.01). Serum urea nitrogen of 150 mg/kg SPM group and 300 mg/kg SPM group were significantly lower than that of ITM group (*p* < 0.01) (Table 5). Serum glutathione peroxidase activity of 300 mg/kg SPM group was increased, compared to that of 150 mg/kg SPM group and 500 mg/kg SPM group (*p* < 0.01) (In Table 6). Serum CAT activity of 300 mg/kg SPM group was also significantly greater than that of 150 mg/kg SPM group (*p* < 0.05), but not different between the other groups (*p* > 0.05). Among all groups, Cu/Zn SOD activity of the liver was lowest in the ITM group (*p* < 0.01). The CAT activity of the liver in 150 mg/kg SPM group was significantly higher than those in ITM and 300 mg/kg SPM groups (*p* = 0.01).

**TABLE 5:** Effect of supplementation of ITMs and SPMs in basal rations on serum biochemical of chickens.

Item	1,000 mg/kg ITM	150 mg/kg SPM	300 mg/kg SPM	500 mg/kg SPM	SEM	*p*-value
Albumin, g/L	13.03	15.65	15.24	14.34	0.44	0.19
Alkaline phosphatase, King’s unit/100 ml	24.65	29.30	29.15	27.36	1.95	0.84
Total cholesterol, mmol/L	3.10^a^	1.36^b^	1.69^b^	1.85^b^	0.11	<0.01
Urea nitrogen, mmol/L	7.2^a^	3.63^b^	3.5^b^	5.39^ab^	0.29	<0.01
Total protein, ug/ml	23.81	23.58	23.03	26.62	0.97	0.59

Numbers in the same row followed by different letters (a,b) are statistically different (*p* < 0.05).

**TABLE 6:** Effect of supplementation of ITMs and SPMs in basal rations on serum and liver antioxidant parameters of chickens.

Item	1,000 mg/kg ITM	150 mg/kg SPM	300 mg/kg SPM	500 mg/kg SPM	SEM	*p*-value
Serum antioxidant parameters						
MDA, nmol/ml	2.86	2.62	2.52	2.62	0.15	0.90
T-AOC, U/ml	0.52	0.49	0.44	0.46	0.01	0.18
Cu/Zn SOD, U/ml	196.93	181.17	185.58	175.40	3.25	0.16
GSH-Px, U/ml	1,260.12^ab^	965.50^c^	1,356.51^a^	1,044.01^bc^	25.57	<0.01
CAT, U/ml	10.37^ab^	8.05^b^	12.99^a^	10.19^ab^	0.51	0.02
Liver antioxidant parameters						
MDA, nmol/mgprot	0.30	0.39	0.35	0.35	0.02	0.56
T-AOC, mmol/mgprot	0.04	0.04	0.04	0.04	0	0.14
Cu/ZnSOD, U/mgprot	212.93^b^	284.49^a^	288.94^a^	288.64^a^	5.03	<0.01
GSH-Px, U/mg	39.58	43.05	41.69	45.05	1.05	0.30
CAT, U/mgprot	11.17^b^	15.57^a^	12.16^b^	13.35^ab^	0.37	0.01

Numbers in the same row followed by different letters (a,b,c) are statistically different (*p* < 0.05).

### Mineral deposition in tibia

**FIGURE 1:**
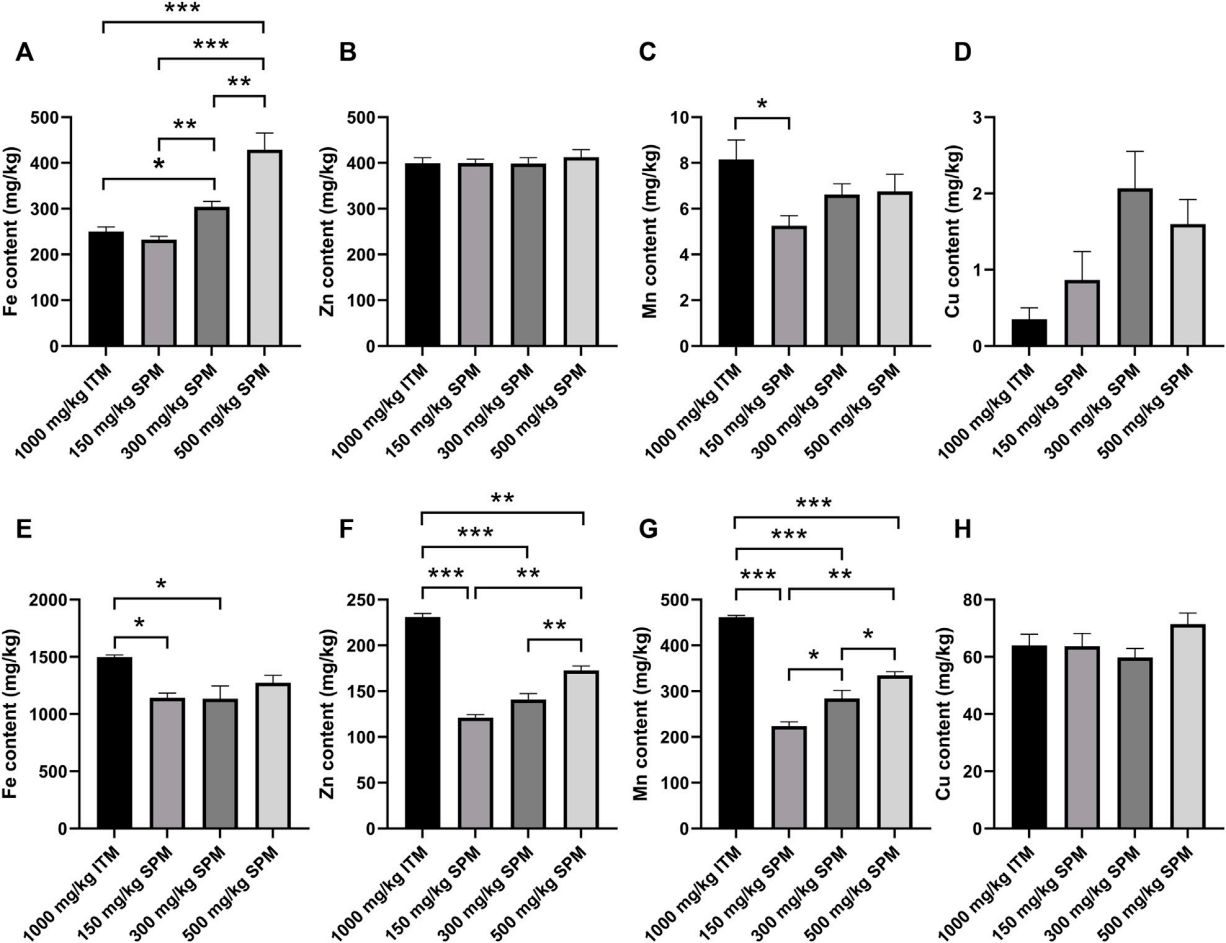
Effect of supplementation of ITM and SPM in basal rations on tibial mineral deposition and fecal mineral excretion of chickens. The deposition concentrations of iron, zinc, manganese, and copper in tibial ash were **(A–D)**, respectively. The fecal excretion concentrations of iron, zinc, manganese, and copper were **(E–H)**, respectively. Asterisks indicate statistically significant differences. * indicates *p* < 0.05; ** indicates *p* < 0.01; *** indicates *p* < 0.0001. Data are expressed with mean ± SEM.

Among the three SPM supplementation groups, the iron contents in the tibia increased with increasing supplementation amount. The iron content of both 500 mg/kg SPM and 300 mg/kg SPM group were significantly higher than that of ITM group (*p* < 0.0001 and *p* < 0.01, respectively) (Figure 1A). Manganese content in the tibia of 150 mg/kg SPM group was significantly lower than that of ITM group (Figure 1C). There were no significant difference in the content of zinc and copper in tibial ashes among all groups (*p* > 0.05) (Figures 1B,D).

### Mineral content in feces

Fecal iron content were significantly reduced in both 150 mg/kg SPM group and 300 mg/kg SPM group (*p* < 0.05), compared with ITM group (Figure 1E). The content of both zinc and manganese were significantly reduced in SPM groups (*p* < 0.01), compared with ITM group (Figures 1F,G). Copper content, on the other hand, were not affected by different sources of supplements (Figure 1H).

## Discussion

Growth parameters are usually used to evaluate the growth performance of broiler chickens. And slaughter parameters can reflect the meat production performance of poultry, which is an important economic indicator. Our study observed no significant differences on growth performance of chickens supplemented with inorganic and organic micro-nutrient supplements. Similar results was observed by Zhu et al. (2019), who reported that replacing inorganic trace minerals with reduced levels chelated trace minerals (30% and 50% of inorganic trace minerals levels) had not affect growth performance of broiler chickens. The authors mentioned that advanced chelation technology could reduce the supplementation amount of trace minerals in broilers because of higher bio-availability of trace minerals in the chelates. The results also agreed with De Marco et al. (2017) who showed that reduced supplementation of copper, iron, zinc, and manganese (50% of the commercial dose recommendation) in the form of glycine metal chelate hydrate, or amino acid metal chelate hydrate had no significant adverse effects on poultry growth. Although many studies had shown that low levels of organic trace minerals could replace inorganic trace minerals without harmful effects on birds, some studies had results different from ours. Sirri et al. (2016) in a 51-day broilers study found supplementation with organic trace minerals significantly improved growth and feed conversion ratio. Singh et al. (2015)’s research revealed that supplementation of broilers with methionine chelates or yeast proteinate forms of Cu, Fe, Mn, and Zn improved body weight and feed conversion ratio and markedly reduced content of the trace elements. The different results may be due to different sources, the supplementation level, the characteristics of the broiler breed, as well as the age, feeding environment, feeding practices, etc. Each of these influencing factors has the potential to make a difference in the therapeutic effect on birds (Ghasemi et al., 2020). In this study, no significant differences were observed in feed intake, body weight gain, etc. The reason may be that the high bioavailability of SPM already met the nutritional requirements of the poultry.

In this study, dietary supplementation with low doses of organic trace minerals did not affect the dressing percentage, semi-eviscerated weight, eviscerated weight, breast muscle percentage, leg muscle percentage, and abdominal fat percentage compared with the inorganic supplementation group. The results agreed with the study by Sirri et al. (2016) who reported supplementation with organic trace minerals did not affect carcass traits. However, other studies have reported different results on carcass traits with the addition of organic trace minerals. El-Hussein et al. (2012) reported that supplementation of experimental diets with 50% Zn, 50% Mn, and 50% Cu organic trace elements (50% of commercially recommended ITM levels) in 35-day-old Arbor Acres broilers significantly increased carcass and breast muscle production. In a study by Zhao et al. (2010), supplementation of a mixture of inorganic and chelated trace minerals (50:50) to the diet increased pectoral muscle production in male broilers (Cobb 700) compared to 100% inorganic supplements, but it did not affect carcass traits in 52-day-old ROSS 308 broilers. Kwiecień et al. (2016) demonstrated that use of 50 and 25 mg/kg of zinc (Zn-Gly chelate), which are lower than the recommended dose, increased the weight of breast and leg muscles, but no difference was observed regarding the slaughter weight of chickens.

Serum biochemical parameters are key indicators that can reflect the animal’s nutritional status. The supplemented trace elements (copper, iron, zinc, and manganese) are related to lipid metabolism. In animal organisms, zinc is involved in skeletal formation, cell-mediated immunity, systemic host defense, sexual maturation, and tissue growth (Lü and Combs, 1988). Previous studies have claimed that the addition of different concentrations of inorganic zinc can slightly reduce serum cholesterol levels (Uyanik et al., 2001). Lü and Combs (1988) reported that providing different amounts of zinc in the diet did not affect serum cholesterol levels. In our study, feeding organic trace minerals to broilers was more effective in reducing total cholesterol levels than providing inorganic forms. The level of urea nitrogen in the blood is an essential measure of kidney function. Therefore, people often assess the health of the kidney by looking at the level of urea nitrogen in the blood. In our study, chickens fed organic supplements had significantly lower uric acid levels than inorganic treatments, suggesting that dietary supplementation with organic trace elements had a beneficial effect on kidney function.

Excessive free radical production causes damage and injury to cells, resulting in oxidative stress. GSH-Px and Cu/Zn SOD, CAT are the main enzymes of the antioxidant enzyme system. They act as enzyme scavengers for free radicals, thus protecting cells from damaging processes and alleviating oxidative stress promptly. The group of 300 mg/kg SPM had the highest activity of GSH-Px and CAT among the four treatments, but they were not significantly different from the ITM group. In assessing the antioxidant status of broilers, 300 mg/kg SPM had the highest activity of GSH-Px and CAT among the four treatments. However, 150 mg/kg SPM group chickens had the lowest GSH-Px activity among all groups. The activity of Cu/Zn SOD in these chickens remained relatively stable. In the current study, dietary supplementation with organic trace minerals significantly increased Cu/Zn SOD in the liver. In previous studies of dietary supplementation with individual organic complex minerals, organic zinc and copper promoted the synthesis of Cu/Zn SOD, thereby increasing the activity of this enzyme (Sahin et al., 2005; Sun et al., 2011) which are consistent with our results.

In current study, feeding organic trace elements increased the iron content in tibiae. Except for the 150 mg/kg SPM treatment group, the addition of SPM significantly increased iron content in the tibia compared to the ITM group. Our results are similar to those reported by Cao et al. (1996). Similarly, it has also been reported that the addition of iron chelated with glycine increased in iron content in broiler tibiae (Ma et al., 2012). On the other hand, the manganese content of tibial ash was significantly reduced in the 150 mg/kg SPM group, and no differences were observed between the other two organic groups and the control group. Aksu et al. (2011) showed the similar results. The concentration of zinc in the tibia is a predictor of good growth in poultry because good tibial development is related to the growth and development of the animal organism (Bao et al., 2007). In the present study, there was no significant difference in zinc content among the treatment groups which also prove that growth performance was unaffected. The Cu concentration of each treatment group in the tibia did not show any difference and was not affected by treatments. Most of the copper is stored in the liver and then released into the bile and blood. For the most part, bone is not considered the primary site of copper storage (Spears 1999), so chicken tibia copper concentration may not be used as a reliable indicator of bone mineralization (Singh et al., 2015).

Bao et al. (2007) reported that feeding high levels of organic complexes (Cu, Mn, Zn) did not promote growth in broilers and resulted in significant excretion of minerals. Nollet et al. (2008) fed birds 67% and 100% organic rations did not reduce excretion of minerals. Therefore, supplementing the diet with high levels of organic minerals makes the animal “overloaded.” It poses a potential threat to environmental pollution and waste of resources. Reducing micro-nutrient intake through dietary control reduces the heavy metal overload in the fields. This strategy remains an effective and viable solution to mitigate mineral pollution. Our study provided lower levels of trace minerals. In our study, the treatment group with dietary supplementation with organic chelates had significantly lower mineral content, specifically in zinc and manganese, without affecting the growth performance. Supplementation of broilers with 150 mg/kg and 300 mg/kg of organic supplements also effectively reduced iron content. It is possible that, there is a unique transport system enabling minerals, in the form of cyclic structure of the chelates, effectively passing through the intestinal wall, resulting in a higher bioavailability. Our study is consistent with previous studies showing that low doses of organic forms of supplementation not only had no negative impact on growth performance but also effectively mitigated environmental stress (Aksu et al., 2011).

## Conclusion

In our study, replacing inorganic trace mineral supplements with a low dose of small peptide chelated minerals did not negatively affect the performance of 817 white feather broilers. It improved the activity of some serum biochemical and liver antioxidant parameters. It also improved iron deposition in the tibia and significantly reduced mineral content in feces.

## Data Availability

The original contributions presented in the study are included in the article/supplementary material, further inquiries can be directed to the corresponding authors.
